# Growth hormone therapy in children with temple syndrome: the first retrospective study in China

**DOI:** 10.1186/s12887-026-06898-0

**Published:** 2026-04-23

**Authors:** Xiou Wang, Ruimin Chen, Zhe Su, Xin Fan, Lusheng Shen, Fuying Song, Ziqin Liu, Chunxiu Gong, Binyan Cao

**Affiliations:** 1 Department of Endocrinology, Capital Center for Children’s Health, Capital Medical University, Capital Institute of Pediatrics, 2#Yabao Rd, Chaoyang District, Beijing, 100020 China; 2 Department of Endocrinology, Fuzhou Children’s Hospital of Fujian Medical University, Fuzhou, 350005 China; 3 Department of Endocrinology, Shenzhen Children’s Hospital, Shenzhen, 518026 China; 4 Department of Pediatrics, Second Affiliated Hospital of Guangxi Medical University, Nanning, 530007 China; 5 Department of Pediatrics, The First Hospital of Handan, Handan, 056002 China; 6 Department of Endocrinology, Genetics and Metabolism, Beijing Children’s Hospital, Capital Medical University, National Center for Children’s Health, 56#Nan Lishi Rd, Xicheng District, Beijing, 100045 China

**Keywords:** Temple syndrome, TS14, China, UPD(14)mat, Growth hormone therapy, Precocious puberty, SGA

## Abstract

**Background:**

Temple syndrome (TS14) is a rare imprinting disorder caused by dysregulation of imprinted genes in the 14q32 region. Although several cohorts have been reported, pediatric data from China are still lacking. This study aimed to characterize the clinical features in Chinese children with TS14 and evaluate their response to recombinant human growth hormone (rhGH), including outcomes associated with initiating treatment before two years of age.

**Methods:**

A multicenter retrospective review was conducted involving seven children diagnosed with TS14 across six pediatric centers in China. Clinical characteristics, growth parameters, genetic testing, and treatment outcomes were extracted from medical records. Molecular diagnosis was established by methylation-specific assays, with additional molecular-genetic testing performed as indicated. Changes in height standard deviation score (SDS) were analyzed descriptively.

**Results:**

Seven patients (1 male, 6 females) were included, with a median age at diagnosis of 2.0 (IQR: 0.67–7.33) years. Maternal UPD(14) was confirmed in one case and strongly suspected in three cases, whereas in the remaining three cases the underlying mechanism could not be determined. Most patients were small for gestational age (71.4%) and all exhibited growth retardation, delayed language development, dysmorphic features, with feeding difficulties (85.7%), and hypotonia (71.4%). All three older children subsequently developed central precocious puberty. Six patients who were initiated on rhGH therapy before puberty (median age at initiation, 2.42 years), achieved a mean height SDS increase of 1.19 after one year. Among the three patients treated before age two, the mean height SDS gain was 1.15, with caregiver-reported improvements in motor and language development, and no adverse events reported.

**Conclusions:**

This inaugural TS14 cohort from China shows clinical features largely consistent with those reported in international cohorts, with a high frequency of precocious puberty among older patients. rhGH therapy yielded substantial short-term gains in height, and initiation of treatment before age two was accompanied by caregiver-reported developmental improvements, although standardized developmental assessments were not available. Early genetic diagnosis and timely intervention are essential, while long-term outcomes require further study.

## Background

 Temple syndrome (TS14, OMIM #616222) is a rare imprinting disorder resulting from dysregulation of both maternally and paternally expressed genes located within the 14q32 imprinted region [1]. Under normal physiological conditions, the paternal allele within the 14q32.2 domain expresses protein-coding genes such as *DLK1*, *RTL1*, and *DIO3*, whereas the maternal allele expresses long non-coding RNAs (lncRNAs) including *MEG3/GTL2*, *RTL1as*, *MEG8*, as well as multiple short non-coding RNAs (ncRNAs) [2]. Molecular abnormalities such as maternal uniparental disomy of chromosome 14 (UPD(14)mat), imprinting defects, or paternal deletions, lead to the absence or downregulation of paternal gene expression, thereby leading to the development of TS14 [1–4].

The clinical phenotype of TS14 is primarily attributed to functional abnormalities of the paternally expressed genes *DLK1* and *RTL1*. The *DLK1* gene plays a pivotal role in somatic growth, neural and muscular development, and the regulation of the Notch signaling pathway. It also suppresses preadipocyte differentiation and influences both feeding regulation and pubertal onset [5]. The *RTL1* gene is essential for placental development, somatic growth, and muscle function [6]. Clinically, TS14 is characterized by small for gestational age (SGA), postnatal growth retardation, feeding difficulties, hypotonia, motor and language delays, truncal obesity, and mild craniofacial dysmorphisms [1–4]. Rapidly progressive central precocious puberty (CPP) is a distinctive endocrine hallmark, often leading to accelerated bone maturation and compromised adult height. The clinical phenotype overlaps considerably with that of other imprinting disorders, particularly Prader–Willi syndrome (PWS) and Silver–Russell syndrome (SRS), which contributes to diagnostic uncertainty and delays [2–4].

Over the past decade, multiple cohorts from Japan and Western countries have expanded the understanding of TS14 [3, 4, 7, 8]. However, no pediatric cohort studies have been reported from China, and existing literature is limited to isolated case reports. This gap hampers our understanding of whether Chinese children exhibit unique phenotypic patterns, encounter specific diagnostic challenges, or differential responses to treatment.

Growth hormone (GH) therapy has shown beneficial effects in TS14. Previous studies demonstrate that recombinant human GH (rhGH) improves short-term linear growth and may enhance long-term height outcomes [9, 10]. This is particularly relevant for TS14, as growth failure begins in infancy, while unrecognized or untreated CPP may impair height gains later in childhood [11]. However, evidence regarding the developmental benefits of initiating GH therapy at a very early stage, as well as its interaction with precocious puberty, remains limited.

To address these gaps, we conducted the inaugural multicenter clinical characterization of TS14 in Chinese children, summarizing clinical features, endocrine manifestations, and responses to rhGH therapy, including outcomes of early treatment. This study provides the first foundational dataset from China, facilitating enhanced recognition, diagnosis, and clinical management of TS14 within the Chinese pediatric population.

## Methods

### Participants

This multicenter retrospective study included seven pediatric patients diagnosed with TS14 between April 2021 and March 2025 across six tertiary pediatric centers in China. Two were from the Capital Center for Children’s Health and the remaining five were each from the following centers: Beijing Children’s Hospital; Shenzhen Children’s Hospital; Fuzhou Children’s Hospital; the Second Affiliated Hospital of Guangxi Medical University; and Handan First Hospital. All patients exhibited a spectrum of manifestations consistent with TS14 (including SGA, growth retardation, developmental delay, early-onset obesity, and precocious puberty), with molecular confirmation of the diagnosis. Medical records were reviewed to obtain demographic information, perinatal history, anthropometric measurements, developmental and endocrine features, treatment regimens, and follow-up data. This study was approved by the Ethics Committee of the Capital Center for Children’s Health, Capital Medical University (SHERLLM2025055).

### Genetic testing

Given the broad phenotypic overlap between TS14 and other imprinting disorders, all patients underwent a stepwise genetic workup. In several cases, initial evaluations included whole-exome sequencing (WES), copy-number variation (CNV) analysis, and/or methylation testing to differentiate TS14 from alternative etiologies, such as SRS or PWS. These assessments did not identify alternative etiologies explaining the observed phenotype. A molecular diagnosis of TS14 was established in all patients by methylation-specific multiplex ligation-dependent probe amplification (MS-MLPA) targeting the 14q32 imprinted region, demonstrating an abnormal methylation pattern consistent with TS14. Where available, additional analyses (e.g., CNV-seq Plus with MS-qPCR or WES-derived SNP/LOH analysis) were used to support the underlying mechanism: UPD(14)mat was confirmed in one case, strongly suspected in three cases, whereas in the remaining three cases the underlying mechanism could not be definitively differentiated from an imprinting/methylation defect.

### Clinical assessments

Data collected included gender, age, maternal pregnancy history, birth details, clinical manifestations, medication use, and treatment follow-up. Growth parameters were systematically recorded through December 2025. Laboratory evaluations included a complete blood count, assessments of liver and kidney function, thyroid function testing, blood lipid profiling, glycated hemoglobin, insulin, insulin-like growth factor 1 (IGF-1), and sex hormone levels. Imaging studies performed included bone age, pituitary magnetic resonance imaging (MRI), echocardiography, and abdominal ultrasonography. SGA was defined as birth weight below the 10th percentile (P10) for gestational age based on the Chinese neonatal growth reference standards [12]. Patients’ growth parameters were further evaluated using growth charts based on the Chinese population [13].

### Statistical analysis

All data were obtained from standardized electronic medical records. Due to the limited sample size, only descriptive statistics were employed. Continuous variables with non-normal distributions are presented as median (IQR), while categorical variables are expressed as percentages. No inferential statistical analyses were conducted.

## Results

### Clinical characteristics

Seven children with TS14 (1 male and 6 females) were included. The median age at diagnosis was 2.0 (IQR: 0.67–7.33) years. Perinatal and baseline characteristics are summarized in Table 1. Two patients (28.6%) were preterm and appropriate for gestational age (AGA), while five (71.4%) were full-term but SGA.

**Table 1:** Clinical characteristics of Chinese patients with temple syndrome

Variables	Case 1	Case 2	Case 3	Case 4	Case 5	Case 6	Case 7
Sex	Female	Male	Female	Female	Female	Female	Female
Age at Diagnosis (years)	7.33	0.5	0.67	1.25	2	5.33	8.25
Gestational Age (weeks)	35	35+ 1	38+ 5	40+ 2	37	Term(37–42)	38
Delivery	Cesarean	Cesarean	Cesarean	Cesarean	Vaginal	Vaginal	Vaginal
Birth Weight (g)	2250	2090	2350	2150	2300	1900	2000
Birth Height (cm)	43	44	NA	45	44	40	NA
Perinatal Abnormalities	-	Postnatal asphyxia	Transient neonatal hypoglycemia	-	-	Oligohydramnios, postnatal asphyxia	-
Feeding Difficulties	+	+	+	+	-	+	+
Hypotonia	+	+	+	-	-	+	+
Motor Development Delay	+	+	-	+	-	-	+
Language Development Delay	+	+	+	+	+	+	+
Growth Retardation	+	+	+	+	+	+	+
Malnutrition at Diagnosis	-	-	-	+	+	-	-
Obesity	+	None to date	None to date	None to date	None to date	None to date	None to date
Precocious Puberty	6.8 years menarche	None to date	None to date	None to date	None to date	5 years breast development	8 years menarche
Facial Features	Round face, low nasal bridge, small hands and feet	Low nasal bridge, micrognathia	Triangular face, relatively large head, micrognathia, low nasal bridge, short philtrum, prominent ears	Triangular face, micrognathia, frontal prominence, wide nasal tip, upturned nose	Frontal prominence, crowded facial features, upturned nose, high-arched palate, small hands and feet	Ptosis, low nasal bridge, high-arched palate	Epicanthal folds, high-arched palate, shield chest
Age at rhGH initiation (years)	3	1.25	1	1.83	3.5	4.83	8.42
Duration of rhGH therapy (years)	1	1.08	1	1	1.5	3.5	2.17
First-year growth velocity (cm/year)	12	13.5	14	11	9.8	12	5.9
Height Before Treatment (SDS)	-3.58	-3.72	-3.04	-2.43	-4.13	-3.44	-0.7
IGF-1 (ng/mL)	299	76.2	51.2	29	71.1	113	436.8

*Abbreviations: NA* not available

All patients presented with postnatal growth retardation and delayed language development. Dysmorphic facial features were observed in all cases, including a broad forehead, short palpebral fissure length, epicanthus, a short nose with anteverted nares, a long philtrum, an everted upper lip, downturned mouth corners, and a high-arched palate. Feeding difficulties were reported in six patients (85.7%), hypotonia in five (71.4%), and mild motor delay in four (57.1%).

Three patients (Cases 1, 6, and 7) developed CPP. Two were diagnosed based on early menarche, while Patient 6 developed breast enlargement, gonadal enlargement, accelerated growth, advanced bone age, and elevated pubertal hormone levels during GH treatment, confirming CPP. Bone age advancement ranged from + 1 to 3.5 years. One older patient developed truncal obesity after the age of five and, by seven years of age, met the criteria for prediabetes (HbA1c 6.3%) with hyperinsulinemia and metabolic dysfunction-associated steatotic liver disease (MASLD). Brain MRI was normal in all patients except Case 6, who showed hypomyelination and a pineal gland cyst. No additional abnormalities were detected on laboratory or imaging examinations.

### Recombinant human growth hormone therapy

**Fig. 1:**
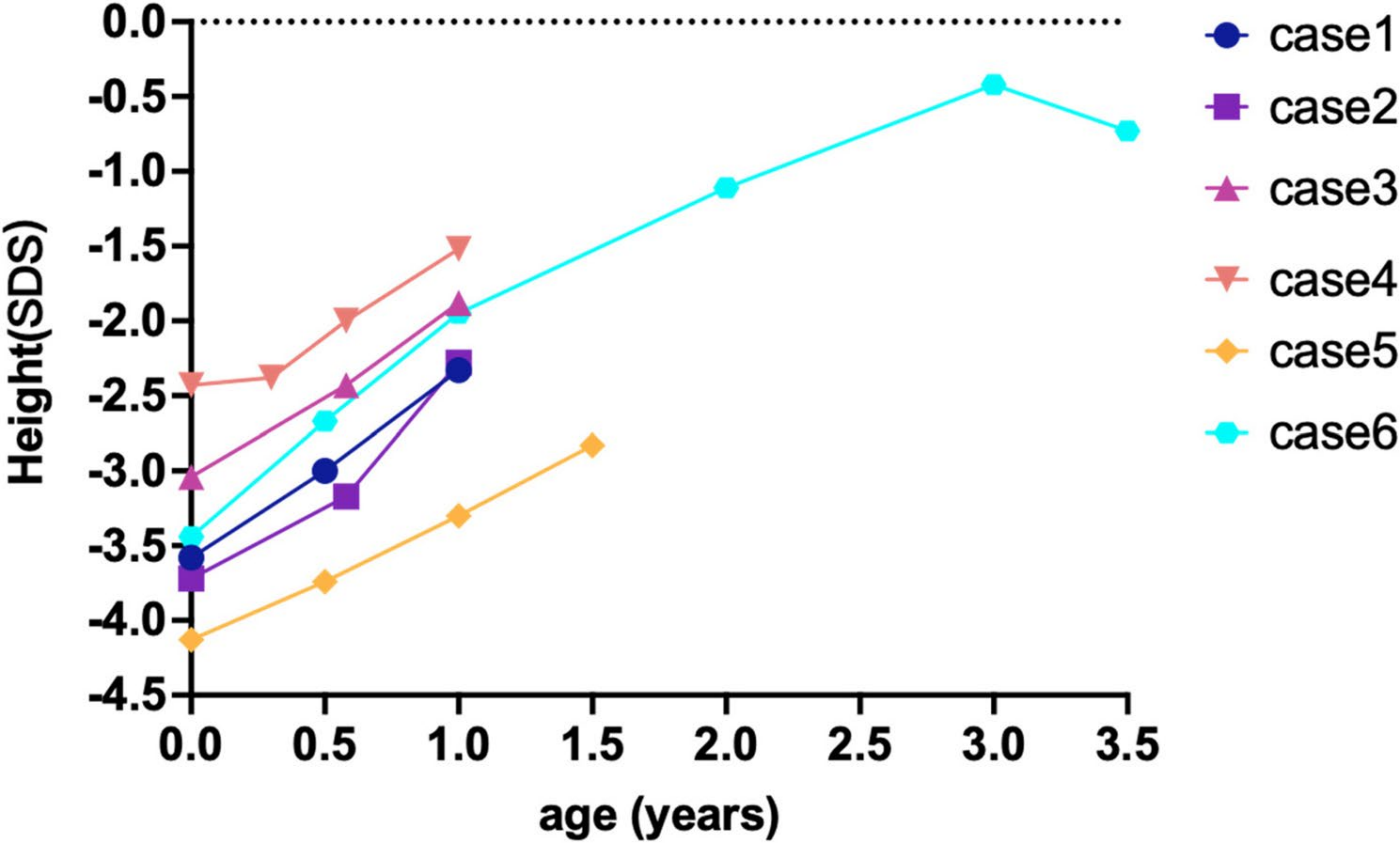
Height SDS improvement in TS14 patients (cases 1–6) after rhGH treatment; Height SDS decline in case 6 after combined treatment with GnRHa

Six patients initiated rhGH therapy before puberty at a median age of 2.42 years (IQR: 1.25–3.5). The median treatment duration was 1.04 years (IQR: 1–1.5). Four patients received daily short-acting rhGH at 0.13–0.20 IU/kg/day, and two patients received long-acting rhGH at 0.14–0.20 mg/kg/week. The baseline height SDS was -3.51 (IQR: -3.72 to -3.04). After one year of therapy, height SDS improved by 1.21 (IQR: 0.90–1.44). First-year growth velocity was 12.0 cm/year (IQR: 11.0–13.4). One patient who continued therapy for 3.5 years maintained growth velocities of 9.3 cm/year in the second year (bone age increased from 5.2 to 9.0 years) and 9.2 cm/year in the third year (bone age, 10.2 years). During follow-up, regular clinical and biochemical monitoring including liver and renal function and fasting glucose showed no significant abnormalities, and no treatment-related adverse events were reported. Details are provided in Table 1. Growth charts are shown in Fig. 1.

### CPP-related therapy

Case 1 showed no signs of pubertal development on examination at 5 years. She presented at 6.8 years with menarche. Although the onset of breast development was not documented, the interval between these visits suggests rapid pubertal progression. She started GnRHa therapy at 7.3 years (bone age advanced by 3.5 years), followed by combined rhGH therapy at 7.75 years.

Case 6 began rhGH at 4.83 years of age, developed breast enlargement at five years, and started GnRHa therapy at 7 years, when bone age was advanced by 2.2 years.

**Fig. 2:**
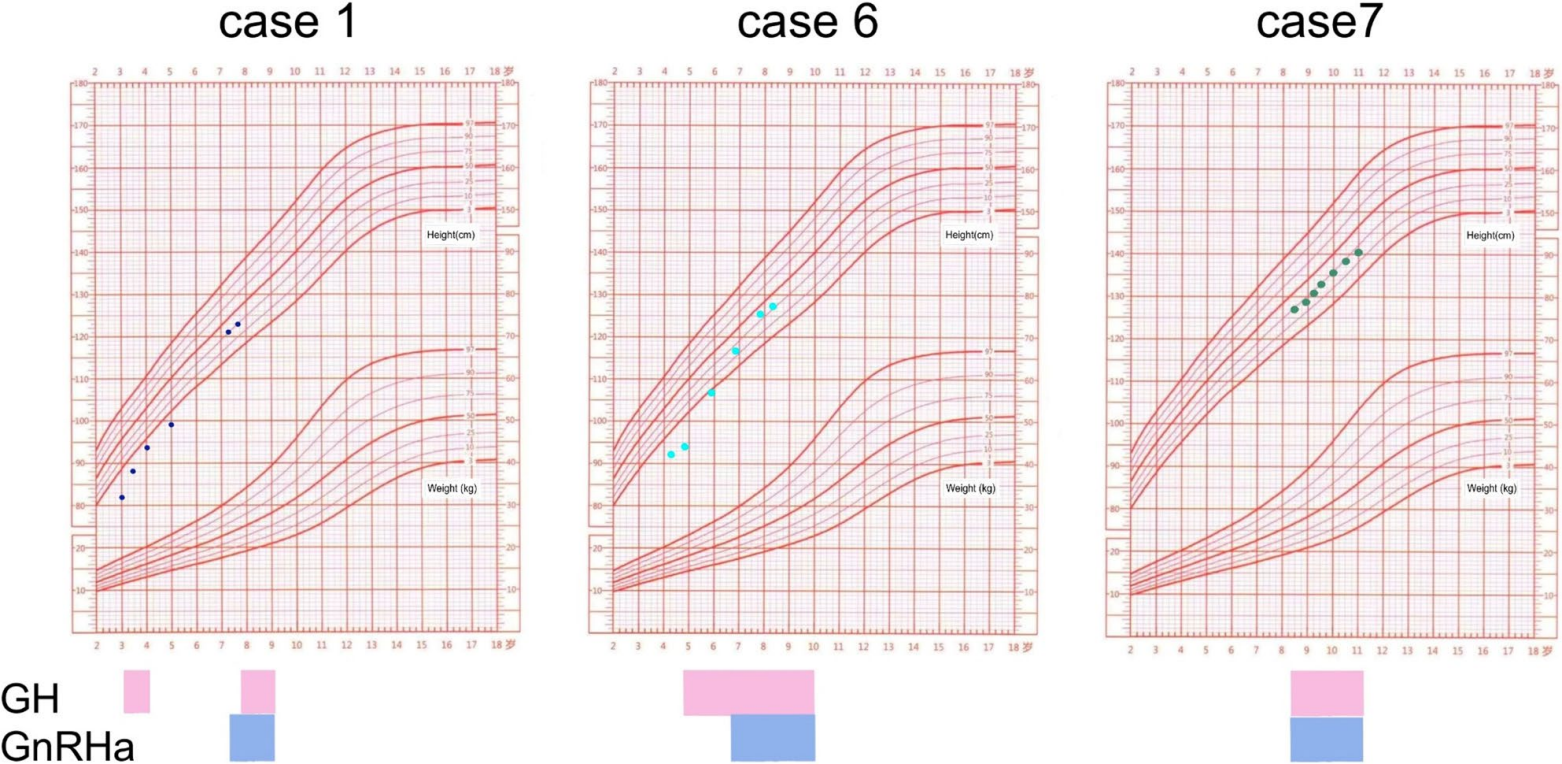
Growth trajectories of three TS14 patients with CPP who received combined rhGH and GnRHa treatment. The pink area indicates the period of rhGH therapy, and the blue area indicates the period of GnRHa therapy

Case 7 had menarche at 8 years and began combined rhGH and GnRHa therapy at 8.4 years. Minimal improvement in height SDS was observed over two years of combined therapy. Detailed data are shown in Fig. 2.

### Early treatment subgroup (<2 years at initiation of rhGH)

Three patients initiated rhGH therapy before the age of two. Their mean height SDS increased by 1.15 during the first treatment year. Caregivers reported noticeable improvements in motor and language development, although standardized developmental assessments were not available.

## Discussion

This study presents the first multicenter pediatric cohort of TS14 in China, offering new clinical insights into its phenotypic spectrum, endocrine features, and response to rhGH therapy. While TS14 is increasingly acknowledged on an international scale, reports originating from China remain limited and primarily consist of isolated case descriptions. Our findings show that Chinese children exhibit a clinical phenotype that aligns broadly with those observed in international cohorts [3, 4, 7, 8]. Notably, three patients in this cohort were diagnosed and initiated rhGH therapy before the age of two, which is relatively uncommon in the existing TS14 literature and provides an opportunity for an initial descriptive assessment of outcomes associated with treatment initiated in infancy.

Although definitive UPD testing was not performed in most patients, the majority of cases in our cohort were suspected to have UPD(14)mat, in line with prior reports supporting UPD(14)mat as the predominant molecular mechanism underlying TS14 [6]. This distribution may be influenced by the diagnostic approach used, as SNP array and copy-number analyses are well suited for detecting UPD, whereas methylation defects require targeted methylation assays [14]. Although phenotypes are generally similar across molecular subtypes, subtle differences have been reported (e.g., a higher prevalence of overweight/obesity in patients with methylation defects) [7]. We did not identify paternal 14q32 deletions; prior studies suggest that complete deletions involving DLK1 may result in a typical TS14 phenotype, whereas exon-limited deletions may present with isolated CPP [15]. Because of limitations in molecular testing, we were unable to perform meaningful comparisons across molecular categories, and the incomplete molecular characterization may limit the generalizability of our findings. Larger prospective cohorts with comprehensive molecular profiling are needed to clarify genotype/epigenotype-phenotype correlations and to inform subtype-specific surveillance.

All patients exhibited disease-related features from birth; 71.4% were SGA, and 28.6% were born preterm but AGA. These findings support the notion that TS14 primarily affects fetal growth during late gestation. Postnatal catch-up growth is typically absent, and nearly 90% of children with TS14 develop short stature by two years of age as documented in published cohorts [3]. Although early puberty may transiently normalize height during mid-childhood, accelerated bone maturation ultimately results in reduced adult stature. This is underscored by a reported case of untreated TS14, in which the individual achieved an adult height of 141 cm (–4.76 SDS) [11], highlighting the critical importance of early clinical intervention.

Among the six patients who initiated rhGH therapy during the prepubertal period, short-term height responses were favorable. These findings align with previous reports showing a median first-year gain of 1.31 SDS in TS14, which is comparable to other SGA populations [9]. Long-term benefits have also been documented, with a median adult height SDS of 0.67 observed in a small treated cohort [10] and an approximate increase of 1 SDS reported in Japanese patients [3]. Together, these findings bolster the evidence for sustained growth improvement associated with rhGH therapy. Accordingly, most patients initiated rhGH therapy after the age of two, in accordance with SGA guidelines that recommend treatment when height remains below − 2.5 SDS in the absence of catch-up growth [16]. Given that catch-up growth is rarely observed in TS14, three patients in our cohort began rhGH before age two following early diagnosis. Beyond height gains, caregivers reported improvements in motor function, language development, and muscle tone, with no reported adverse events. Previous studies have similarly reported favorable changes in body composition following rhGH therapy [10]. Evidence from PWS, a disorder that shares clinical features with TS14 such as feeding difficulties, hypotonia, and developmental delay, suggests that rhGH can enhance growth, BMI, cognition, and motor skills in infants and young children [17]. Collectively, these findings suggest potential benefits of earlier rhGH initiation in TS14; however, developmental outcomes require standardized assessment, extrapolation from related disorders should be cautious, and longer follow-up is needed to determine effects on final adult height.

All three older patients in this cohort developed rapidly progressive CPP, a characteristic endocrine feature of TS14. Accelerated bone maturation may hinder adult height regardless of treatment. In a Japanese cohort, height SDS declined after 4–6 years of therapy, likely reflecting the onset of CPP [3]. Similarly, two patients in this study (Cases 1 and 7) were diagnosed late and initiated combined rhGH and GnRHa therapy only after menarche, resulting in suboptimal height improvement. These findings emphasize the importance of early initiation of rhGH and diligent monitoring for precocious puberty to facilitate timely GnRHa intervention.

Beyond growth and puberty, feeding difficulties, hypotonia, and language delay were prevalent in this cohort. Hypotonia likely contributes to impaired sucking strength and delayed motor development. Consistent with earlier reports, language abilities showed progressive improvement with age [14]. Mild dysmorphic features were observed in nearly all patients, although their presentation was heterogeneous. Interestingly, several phenotypic features, such as small hands and feet, dental anomalies, and joint hypermobility, were less frequently observed in our Chinese cohort compared with Japanese and Western cohorts [3, 4]. Whether these differences represent potential ethnic variation remains to be determined. Previous studies have reported obesity in 20–33% of patients [3, 7], whereas MASLD has rarely been described. In our cohort, one older patient developed MASLD, suggesting that metabolic abnormalities may represent underrecognized long-term comorbidities warranting ongoing surveillance. Given the role of DLK1 in adipocyte differentiation and metabolic regulation, patients with TS14 may be predisposed to adipose tissue dysfunction and related metabolic disturbances.

The phenotypic overlap between TS14, SRS, and PWS is significant [18]. Many TS14 infants display SRS-like features (prominent forehead, relative macrocephaly), whereas some older children may present with characteristics resembling PWS, including almond-shaped eyes, flat nasal bridge, small hands/feet [3, 4]. Distinguishing features include higher birth weight and a markedly increased incidence of CPP in TS14 relative to SRS [4], along with milder hyperphagia and hypogonadism relative to PWS [19]. Body composition analyses also indicate relatively lower lean mass and normal or increased fat mass in TS14 when compared with SRS [7]. Moreover, prolonged nasogastric feeding, which is common in PWS and SRS, is either uncommon or brief in TS14 infants [20]. Despite these distinguishing traits, many affected children remain undiagnosed until later childhood. TS14 should be considered in children presenting with SGA, postnatal growth failure, hypotonia, or characteristic facial features, particularly when tests for SRS and PWS yield negative results.

The small sample size, attributable to the rarity of TS14, limited statistical power and the generalizability of our findings. Reported developmental changes among patients who initiated rhGH at a younger age were based primarily on clinical observations rather than standardized assessments, which may introduce subjectivity. Future prospective studies should incorporate validated neurodevelopmental tools (e.g., the Bayley Scales of Infant and Toddler Development) to more rigorously quantify motor and language outcomes. Long-term outcomes, including final adult height and metabolic trajectories, were not available. Furthermore, incomplete molecular subtyping and limited representation of molecular categories precluded analyses of genotype/epigenotype–phenotype correlations, which have been explored in larger international cohorts [3, 4, 7]. Nevertheless, the multicenter design and detailed clinical characterization provide useful data on TS14 in Chinese pediatric patients.

In conclusion, this initial pediatric TS14 cohort from China reveals clinical features that align with international findings, including SGA, feeding difficulties, hypotonia, growth retardation, mild dysmorphic features, delayed language development, and frequent CPP in older children. rhGH therapy was associated with short-term gains in height, and earlier initiation was accompanied by caregiver-reported developmental improvements in a small subset. Enhanced awareness, early molecular testing, and timely management of puberty are crucial for improving outcomes in children with TS14 in China. 

## Data Availability

All data supporting the findings of this study are contained within the article.
